# The effect of non-pharmacological interventions on psychological stress and quality of life of parents of children with retinoblastoma: A protocol for systematic review and meta-analysis

**DOI:** 10.1097/MD.0000000000028148

**Published:** 2021-12-23

**Authors:** Lei Wu, Xitao Xiang, Hong Guo, Hui Tan

**Affiliations:** The Central Hospital of Enshi Tujia and Miao Autonomous Prefecture, Enshi, Hubei Province, China.

**Keywords:** anxiety, depression, meta-analysis, non-pharmacological therapies, protocol, quality of life, retinoblastoma

## Abstract

**Background:**

Retinoblastoma is the most common malignant tumor in infancy and early childhood. Due to the high incidences of intracranial metastasis and distant metastasis, retinoblastoma not only threatens the life of affected children, but also brings heavy mental stress to their parents. A strong mental stress often leads to anxiety, depression, and other adverse emotions, which is very unfavorable to the treatment and prognosis by generating great psychological pressure and reducing the quality of life of the family. Reducing the psychological stress of the parents and improving the quality of life of the family are beneficial to the treatment and prognosis of retinoblastoma in children. However, there are no recommended non-pharmacological therapies to reduce the psychological stress and improve the quality of life of the parents of children with retinoblastoma. This study aims to evaluate the effects of non-pharmacological therapies on psychological stress and quality of life of parents of children with retinoblastoma through a meta-analysis, thus providing clinical evidence.

**Methods:**

Randomized controlled trials reporting the effects of non-pharmacological therapies on psychological stress and quality of life of parents of children with retinoblastoma published before 2021 November will be searched in online databases, including the China National Knowledge Infrastructure, Wanfang, Chinese Scientific Journal Database, China Biomedical Literature Database, PubMed, Embase, The Cochrane Library, and Web of Science databases. The Cochrane Quality Assessment Manual will be used to assess the quality of the included literatures. Meta-analysis will be performed using Revman 5.4 software.

**Results:**

This study will evaluate the effects of non-pharmacological therapies on psychological stress and quality of life of parents of children with retinoblastoma via grading anxiety scores, depression scores, and quality-of-life scores.

**Conclusion:**

This study will provide a reliable evidence-based basis for non-pharmacological interventions on parents of children with retinoblastoma.

**Ethics and dissemination:**

Ethical approval was not required for this study. The systematic review will be published in a peer-reviewed journal, presented at conferences, and shared on social media platforms. This review would be disseminated in a peer-reviewed journal or conference presentations.

OSF REGISTRATION NUMBER:

## 1 Introduction

Retinoblastoma is a malignant tumor that originates from photoreceptor precursor cells.^[1,2]^ It is a hereditary disease with familial genetic tendency, which can affect one eye or both eyes, especially in infants and children.^[3–5]^ Because of the treatment difficulty, long treatment period and high medical costs, retinoblastoma poses heavy physical and psychological stress on the parents of affected children.^[6]^ The immense stress often causes a series of adverse emotions like anxiety, tension, and fear in parents.^[7,8]^ Children are young, involuntary and poorly expressed, and they are highly dependent on their parents, whose emotions are transmitted to each other through daily parent–child communication, which in turn affects the treatment and prognosis of the children.^[9–11]^ At present, anti-anxiety and depression drugs can effectively relieve symptoms of anxiety and depression and reduce psychological stress.^[12,13]^ However, long-term use of these drugs can trigger adverse reactions and influence daily life and work at varying degrees.^[14–16]^

Parents of children with retinoblastoma are prone to suffer a series of adverse emotions due to great psychological stress. Accumulating adverse emotions cause disharmonious family atmosphere, declined quality of life, and unwillingness of children and parents to continuous treatment.^[6]^ Although anxiety and depression of patients can be relieved by drugs, adverse emotions will in turn aggravate with the subsided efficacy. Non-pharmacological therapies can effectively change the parents’ misconceptions, expand knowledge of the disease, increase confidence in treatment, relieve mental and psychological stress, and provide more time and energy to accompany their children. It is reported that non-pharmacological therapies like yoga, cognitive-behavioral therapy, aerobic exercise and music therapy can reduce the psychological stress of parents and improve the quality of life of the family, which are beneficial to the treatment and prognosis of retinoblastoma in children.^[17–22]^ Non-pharmacological therapies are not only easy to perform, but also free of adverse effects, which are well suited for clinical application.

However, the effects of non-pharmacological therapies on the psychological stress and quality of life of parents of children with retinoblastoma are unclear. This study aims to evaluate the effects of non-pharmacological therapies on parents of children with retinoblastoma through a meta-analysis, thus providing an evidence-based basis for their clinical application.

## 2 Methods

### 2.1 Protocol register

This meta-analysis protocol is based on the Preferred Reporting Items for Systematic Reviews and meta-analysis Protocols (PRISMA-P) statement guidelines. The protocol of the systematic review was registered on Open Science Framework, and the registration number is DOI 10.17605/OSF.IO/CBWAJ.

### 2.2 Ethics

The data for our study were extracted from published literatures and the recruitment of patients or collection of personal information is not needed. Therefore, ethics committee approval was not required.

### 2.3 Inclusion criteria

1. Randomized controlled trial;
2. Study subjects are parents of children with confirmed retinoblastoma.
3. Interventions: pharmacological therapies applied to control groups and non-pharmacological therapies to observation groups like cognitive-behavioral interventions, music therapy, yoga exercises, massage, etc.
4. Outcome indicators: anxiety score, depression score, quality of life score.

### 2.4 Exclusion criteria

1. Literatures with unavailable full text;
2. Literatures with missing data;
3. Duplicate published literatures.

### 2.5 Searching strategy

Randomized controlled trials reporting the effects of non-pharmacological therapies on psychological stress and quality of life of parents of children with retinoblastoma published before 2021 November will be searched in online databases, including the PubMed, Embase, The Cochrane Library, Web of Science, China National Knowledge Infrastructure, Wanfang, Chinese Scientific Journal Database, and China Biomedical Literature Database. Online searching will be performed by combining MeSH terms and free words. References of eligible literatures will manually be reviewed to prevent missing data. The searching strategy in the PubMed was shown in Table 1.

**Table 1:** Search strategy in PubMed database.

Number	Search terms
#1	Retinoblastoma[MeSH]
#2	Glioblastoma, Retinal[Title/Abstract]
#3	Glioma, Retinal[Title/Abstract]
#4	Neuroblastoma, Retinal[Title/Abstract]
#5	Eye Cancer, Retinoblastoma[Title/Abstract]
#6	Familial Retinoblastoma[Title/Abstract]
#7	Hereditary Retinoblastoma[Title/Abstract]
#8	Sporadic Retinoblastoma[Title/Abstract]
#9	Familial Retinoblastomas[Title/Abstract]
#10	Glioblastomas, Retinal[Title/Abstract]
#11	Gliomas, Retinal[Title/Abstract]
#12	Hereditary Retinoblastomas[Title/Abstract]
#13	Neuroblastomas, Retinal[Title/Abstract]
#14	Retinal Glioblastoma[Title/Abstract]
#15	Retinal Glioblastomas[Title/Abstract]
#16	Retinal Glioma[Title/Abstract]
#17	Retinal Gliomas[Title/Abstract]
#18	Retinal Neuroblastoma[Title/Abstract]
#19	Retinal Neuroblastomas[Title/Abstract]
#20	Retinoblastoma, Familial[Title/Abstract]
#21	Retinoblastoma, Hereditary[Title/Abstract]
#22	Retinoblastoma, Sporadic[Title/Abstract]
#23	Retinoblastomas[Title/Abstract]
#24	Retinoblastomas, Familial[Title/Abstract]
#25	Retinoblastomas, Hereditary[Title/Abstract]
#26	Retinoblastomas, Sporadic[Title/Abstract]
#27	Sporadic Retinoblastomas[Title/Abstract]
#28	OR/1-27
#29	Quality of Life[MeSH]
#30	Life Qualities[Title/Abstract]
#31	Life Quality[Title/Abstract]
#32	Depression[MeSH]
#33	Depressive Symptoms[Title/Abstract]
#34	Emotional Depression[Title/Abstract]
#35	Depression, Emotional[Title/Abstract]
#36	Depressions[Title/Abstract]
#37	Depressions, Emotional[Title/Abstract]
#38	Depressive Symptom[Title/Abstract]
#39	Emotional Depressions[Title/Abstract]
#40	Symptom, Depressive[Title/Abstract]
#41	Symptoms, Depressive[Title/Abstract]
#42	Anxiety[MeSH]
#43	Hypervigilance[Title/Abstract]
#44	Nervousness[Title/Abstract]
#45	Anxieties[Title/Abstract]
#46	OR/29-45
#47	Randomized Controlled Trials as Topic[MeSH]
#48	Clinical Trials, Randomized[Title/Abstract]
#49	Controlled Clinical Trials, Randomized[Title/Abstract]
#50	Trials, Randomized Clinical[Title/Abstract]
#51	Random∗[Title/Abstract]
#52	OR/47-51
#53	#28 AND #46 AND #52

### 2.6 Data screening and extraction

**Figure 1:**
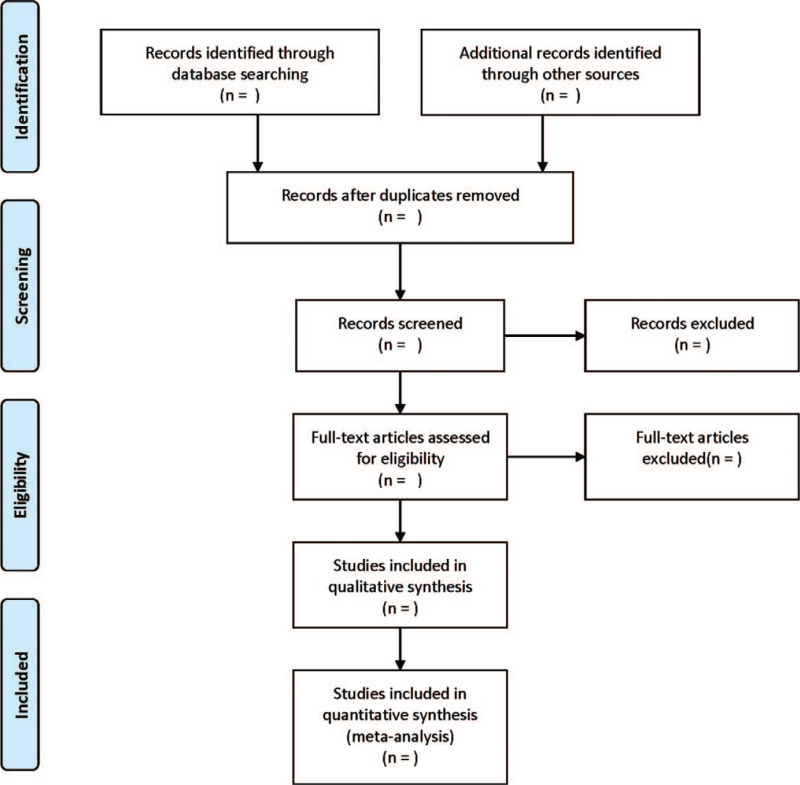
Flow diagram of study selection process.

The retrieved literatures will be summarized and de-duplicated using EndNoteX9. Two researchers will be independently responsible for initial screening according to the inclusion and exclusion criteria by reading the title and abstract, and then re-searched by reading the full text and cross-check. Any disagreement will be dissolved by discussing with the third researcher. The following data will be extracted using a uniform form: first author, country, year of publication, sample size, interventions for control and intervention groups, age, education level, disease stage, and outcome indicators. Missing data will be requested by e-mailing the author; if unavailable, the literature will be excluded. Flow chart of literature screening was shown in Figure 1.

### 2.7 Evaluation of literature quality

The quality of included literatures will be independently evaluated by 2 researchers using the risk of bias assessment tool recommended by the Cochrane Handbook.^[23]^ The following aspects will be evaluated: generation of random sequences, hidden groupings, whether blinding is applied to investigators and subjects, completeness of outcome data, selective reporting, and other sources of bias.

### 2.8 Statistical analysis

#### 2.8.1 Data analysis and processing

Standardized mean differences (SMD) will be calculated for continuous variables, and 95% confidence intervals (95%CI) will be used for all interval estimates. Meta-analysis will be performed using RevMan 5. 4 software, and heterogeneity between included studies will be tested by Chi-square test. *P* ≥ .1 and/or *I*^2^ < 50% suggest no heterogeneity between studies, and a fixed-effects model will be used for the combined analysis; Otherwise, a random-effects model will be used. If *P* < .1 and the source of heterogeneity cannot be determined, meta-analysis will not be performed and descriptive analysis will be used.

#### 2.8.2 Subgroup analysis

Subgroup analysis will be performed according to the treatment stage of children with retinoblastoma and the education level of their parents.

#### 2.8.3 Sensitivity analysis

To test the stability of each combined result of the meta-analysis, sensitivity analysis will be performed by the one-by-one elimination method.

#### 2.8.4 Assessment of reporting biases

A funnel plot was used to assess potential publication bias.^[24]^

## 3 Discussion

Retinoblastoma is the most-common malignant tumor in infancy and early childhood.^[25,26]^ It is prone to intracranial and distant metastasis, which seriously threatens the life and aggravates the mental and psychological stress of their parents.^[27–29]^ Under the long-term heavy pressure, adverse emotions like anxiety, and depression will be continuously aggravated.^[30]^ Parents are the most familiar people to children, and their emotions will directly affect children's emotions and quality of life, which in turn affect the treatment and prognosis.^[11,31–33]^

Non-pharmacological therapies include individualized cognitive behavioral interventions, music therapy, yoga, and massage, etc.^[34]^ They can effectively reduce the generation of adverse emotions, psychological stress and poor outcomes, and improve the quality of life.^[35]^ A growing number of non-pharmacological therapies have been applied to parents of children with retinoblastoma, and achieved good results. However, there is no systematic and comprehensive meta-analysis analyzing the effects of non-pharmacological therapies on parents of children with retinoblastoma. This systematic evaluation will provide an objective evidence-based basis for the implementation of non-pharmacological therapies on the certain population.

However, our study has certain limitations:

1. Included literatures are published in Chinese language, which may have publication bias;
2. The number of included studies is small, and more studies are needed for further analysis;
3. The included population are all from China, which has ethnic variability, and more studies from more countries are needed in the future.

## Author contributions

**Conceptualization:** Hui Tan, Lei Wu.

**Data curation:** Hui Tan, Lei Wu.

**Formal analysis:** Xitao Xiang.

**Funding acquisition:** Hui Tan.

**Investigation:** Xitao Xiang.

**Methodology:** Xitao Xiang.

**Project administration:** Hui Tan.

**Resources:** Xitao Xiang.

**Software:** Hong Guo.

**Supervision:** Hui Tan.

**Validation:** Hong Guo.

**Visualization:** Hong Guo.

**Writing – original draft:** Hui Tan, Lei Wu.

**Writing – review & editing:** Hui Tan, Lei Wu.
